# A Quality Improvement Project on Optimising Intravenous Maintenance Fluid Prescribing in Surgical Patients

**DOI:** 10.7759/cureus.93575

**Published:** 2025-09-30

**Authors:** Jia Xuan Tan, Hao Ming Tan, Kishore Sasapu

**Affiliations:** 1 General Surgery, Diana Princess of Wales Hospital, Grimsby, GBR; 2 General Surgery, Hull University Teaching Hospital, Hull, GBR; 3 Colorectal Surgery, Diana Princess of Wales Hospital, Grimsby, GBR

**Keywords:** hypotonic fluids, intravenous fluid prescribing, isotonic fluids, post-op, surgical patient

## Abstract

Background: Inappropriate intravenous (IV) fluid prescribing is a common and preventable source of morbidity in hospitalised patients. Maintenance fluids should meet daily physiological requirements without causing electrolyte imbalances or fluid overload. National Institute for Health and Care Excellence (NICE) guidelines recommend hypotonic, balanced solutions tailored to patient weight.

Aim: This study aims to evaluate adherence to NICE IV fluid prescribing guidelines in a district general hospital's general surgery department, implement targeted interventions, and assess impact through re-audit.

Methods: A three-phase quality improvement project using the Plan-Do-Study-Act (PDSA) cycle was conducted. Phase 1 involved retrospective analysis of IV maintenance fluid prescriptions for emergency laparotomy patients (n = 21). Phase 2 introduced a new default fluid (0.45% NaCl + 0.15% KCl + 5% dextrose), delivered teaching sessions, and disseminated prescribing guidance. Phase 3 was a re-audit (n = 20) conducted one month post-intervention.

Results: Pre-intervention, only 9.5% of patients received fluids in line with NICE guidance. Sodium and chloride intake exceeded recommended levels by over 200%, while potassium provision was 83% below target. Post-intervention, appropriate prescribing increased to 45%, with reductions in sodium (by 165%) and chloride (by 128%) administration, and improved potassium and dextrose provision.

Conclusion: A multidisciplinary intervention significantly improved adherence to fluid prescribing guidelines, reduced electrolyte excess, and enhanced patient safety. Sustained improvement requires trust-wide policy development, consistent education, and broader implementation across departments.

## Introduction

Intravenous fluids are the most frequently prescribed drug in hospitalised patients, with isotonic saline being the most frequently administered crystalloid fluid [1,2]. However, approximately 20% of patients experience complications due to improper fluid prescribing, such as fluid overload, electrolyte imbalance, and metabolic derangements [3]. Therefore, careful assessment of a patient’s fluid and electrolyte needs is crucial.

There are three primary indications for intravenous fluid administration: maintenance, resuscitation, and replacement [4]. This article focuses specifically on maintenance fluids, which are administered to haemodynamically stable patients to fulfil their daily water, electrolyte, and glucose requirements when oral intake is inadequate or not feasible [4]. Oral hydration remains the most physiological and preferred method for maintaining fluid balance. However, intravenous maintenance fluids are essential in clinical situations where oral intake is limited or contraindicated. Typical examples include patients who are unconscious, those who are nil by mouth (NBM) in preparation for surgery, or individuals with gastrointestinal conditions impairing oral intake.

Fluids can be categorised based on tonicity [5]. Isotonic solutions, such as 0.9% NaCl, Hartmann’s, and Plasma-Lyte, have the same osmotic pressure as plasma and result in no net water movement between compartments. Hence, they distribute primarily in the extracellular compartment [5]. Hypertonic solutions draw water out of cells, causing cellular shrinkage, whereas hypotonic solutions, such as 0.45% NaCl, allow water to move into cells, which may cause cellular swelling. They more closely mimic physiological electrolyte and water requirements, enabling a more balanced distribution between intra- and extracellular spaces [6,7].

Understanding these fluid dynamics is crucial, especially when managing maintenance therapy. Total body water is distributed as two-thirds intracellular and one-third extracellular, with the extracellular compartment comprising one-quarter intravascular and three-quarters interstitial fluid [8]. Due to its isotonic nature, 0.9% NaCl remains largely in the extracellular space, making it suitable for resuscitation but not for maintenance due to its excessive sodium (154 mmol/L) and chloride (154 mmol/L) content, which can lead to hyperchloraemic metabolic acidosis and fluid overload [6].

Conversely, hypotonic fluids such as 0.45% NaCl more closely reflect daily maintenance requirements by allowing more balanced distribution into the intracellular compartment and avoiding excessive electrolyte delivery [6,7].

## Materials and methods

Setting and design

This quality improvement (QI) project was conducted in the general surgical wards at Diana Princess of Wales (DPOW) Hospital located in Grimsby, UK. The audit initiative was led by two Foundation Year doctors who were responsible for designing the data collection tool, gathering data from patient records, and performing the initial analysis. Their involvement ensured that the project reflected the practical challenges faced by junior doctors in fluid prescribing. The project was supervised by a consultant surgeon, who provided oversight on methodology, ensured adherence to clinical governance standards, and guided the interpretation of findings in the context of surgical practice. This collaborative structure not only enhanced the quality and accuracy of the audit but also ensured that the process could be reproduced by similar teams in other institutions.

Problem identification

The project was initiated following informal concerns raised by nursing staff regarding recurrent electrolyte abnormalities, specifically hypokalaemia and hyperchloraemia, among inpatients receiving intravenous (IV) maintenance fluids. Retrospective reviews of blood results indicated that these abnormalities were common and often persisted for several days during inpatient stays. Multidisciplinary discussions, including informal interviews with junior doctors and pharmacists, suggested inconsistencies in fluid prescribing practices. Common issues included the routine use of 0.9% sodium chloride and Plasma-Lyte as maintenance fluids, along with the omission of potassium chloride (KCl) and glucose in daily fluid regimens. Prescribing practices often failed to take into account individual patient factors such as weight and comorbidities, and in many cases, patients were given volumes of fluid that exceeded the recommended daily requirement of 25-30 mL/kg/day.

These practices were noted to diverge from recommendations in NICE guidelines, which state that patients should receive 25-30 mL/kg/day of water, 1 mmol/kg/day of sodium, potassium, and chloride, and 50-100 g/day of glucose to reduce the risk of starvation ketosis [7].

Planning stage

This stage involved defining the primary aims of the project, selecting the clinical setting for implementation, and establishing key dates for project milestones.

Aim

This quality improvement project had three main aims. The first was to assess adherence to the NICE intravenous fluid prescribing guidelines within the general surgery department at our local district hospital [7]. The second was to identify variations in clinical practice and prescribing behaviour. The third was to evaluate the impact of multifaceted interventions, including a change in the default maintenance intravenous fluid and the introduction of educational sessions.

To align with evidence-based guidelines, we introduced a more balanced and commercially available fluid: 0.45% NaCl + 0.15% KCl + 5% dextrose, which contains 77 mmol Na, 97 mmol Cl, 20 mmol K, and 50 g dextrose. This approximates daily requirements more closely than commonly used isotonic alternatives. The selected hypotonic solution ensures balanced electrolyte delivery and provides a glucose source to prevent starvation ketosis and maintain homeostasis in patients not receiving adequate enteral intake.

Baseline audit (Stage 1)

The first stage of the project involved a retrospective audit of maintenance intravenous fluid prescribing in post-operative surgical patients over a five-month period (May to September 2023). Data were collected from general surgical wards, focusing on the type, volume, and electrolyte content of maintenance fluids administered within the first 24 hours post-admission. Fluid requirements were calculated and assessed in relation to individual patient weight.

Inclusion Criteria

The inclusion criteria were adult patients aged 18 years or older who were admitted under the general surgery team, had undergone operative procedures during the study period, and were prescribed maintenance intravenous fluids within the first 24 hours of admission.

Exclusion Criteria

The exclusion criteria were patients with acute kidney injury (AKI), those with heart failure, and those requiring care in high dependency or intensive care units.

These criteria were applied to minimise confounding factors related to altered fluid requirements. The baseline audit provided a detailed overview of existing prescribing practices and identified areas of deviation from established guidelines. It was found that the commonly prescribed fluids for maintenance were Plasma-Lyte and 0.9% sodium chloride. Only 9.5% of patients met the requirement of 25-30 mL/kg/day, with the remainder prescribed more than 30 mL/kg/day. Most patients were administered excessive sodium and chloride and too little or no potassium and dextrose (see Table 1).

Intervention development and implementation (Stage 2)

The second stage involved implementing a series of multifaceted interventions aimed at improving adherence to fluid prescribing standards. The default maintenance fluid was replaced with a commercially available hypotonic solution containing 0.45% NaCl with 0.15% KCl and 5% dextrose. This formulation offers a balanced combination of electrolytes and glucose that aligns more closely with NICE guidelines, prevents starvation ketosis, and facilitates better intracellular distribution.

Secondly, a multidisciplinary educational campaign was launched. The initial audit findings were presented at the local audit meeting. Subsequent teaching sessions were delivered in the surgical department to ensure broad understanding and engagement among surgical doctors. Recognising the importance of continuity in prescribing practices, additional education was also provided to newly rotating resident doctors during their induction periods.

During the initial phases of the intervention, nursing staff expressed concerns regarding the potassium content of the new maintenance fluid. To address these apprehensions and foster confidence in the updated protocol, dedicated teaching sessions were conducted specifically for ward nurses, explaining the rationale and indication behind the fluid selection and reinforcing safe administration practices.

Lastly, an awareness campaign was implemented in the surgical wards. Educational posters detailing appropriate fluid prescribing were displayed on surgical wards, complemented by trust-wide email communications to resident doctors and nursing personnel.

Re-audit (Stage 3)

One month post-intervention (November-December 2023), a re-audit was conducted to assess the impact of the implemented changes. Using the same inclusion and exclusion criteria and data collection parameters as the baseline audit, maintenance fluid prescriptions were analysed to evaluate improvements in compliance with recommended practices and to identify any ongoing challenges.

## Results

A total of 41 patients were included, with 21 in the pre-intervention cohort (Stage 1) and 20 in the post-intervention cohort (Stage 3). The average patient weight was 72 kg in both groups.

**Figure 1:**
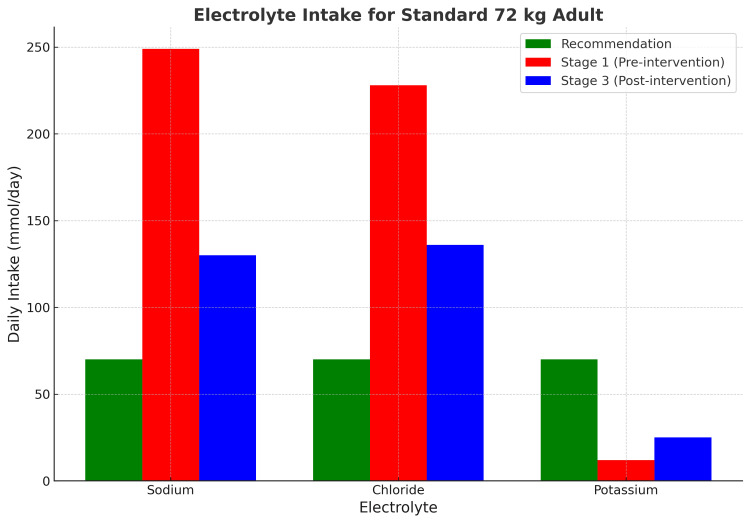
Mean electrolyte concentration for Na, K, and Cl administered

Electrolyte provision also showed improvement, with sodium levels reducing from 249 mmol/day to 130 mmol/day, chloride levels reducing from 228 mmol/day to 136 mmol/day, and potassium increasing from 12 mmol/day to 25 mmol/day (Figure 1 and Table 1).

Sixty-five percent of patients were administered the fluid recommended (0.45% NaCl + 0.15% KCl + 5% dextrose) (Table 1). Glucose provision improved significantly, with only one patient receiving dextrose pre-intervention compared to 13 patients post-intervention, and a mean daily glucose delivery of 49 g (Table 1).

**Table 1:** Comparison of fluid and electrolyte management between Stage 1 and Stage 3

Variables	Category	Normal Range	Stage 1	Stage 3
Number of patients identified		-	21	20
Mean weight (kg)		-	72	72
Average total daily fluid intake (mL)		1800-2160	2500	1932
Mean electrolyte concentration (mmol/day)	Na	72	249 (+246%)	130 (+81%)
	Cl	72	228 (+217%)	136 (+89%)
	K	72	12 (-83%)	25 (-65%)
Dextrose		50-100g/day	Only 1 out of 21 patients received dextrose	13 out of 20 patients received dextrose. Mean dextrose given was 49 g

**Figure 2:**
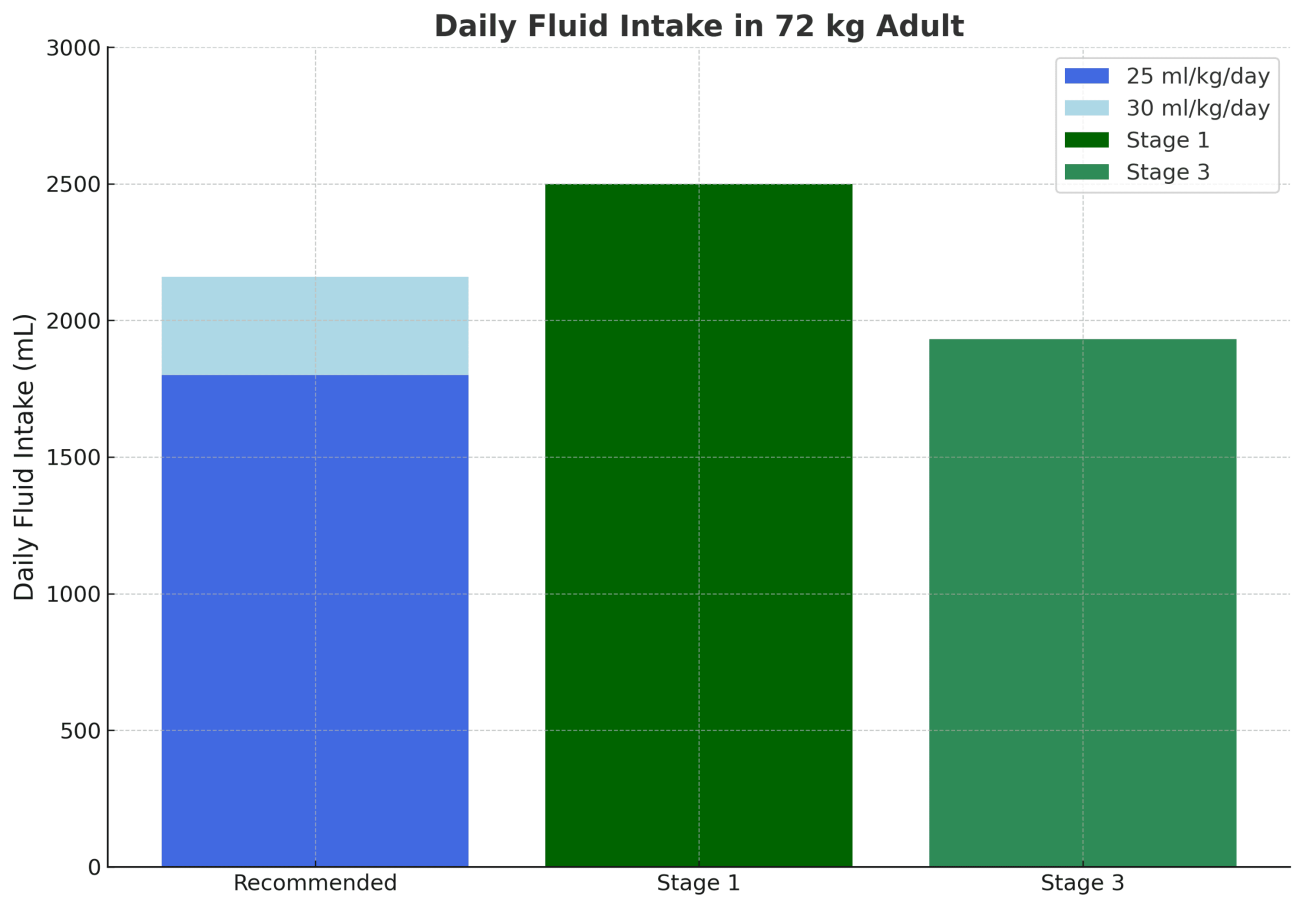
Comparison of mean daily intake of total fluid volume

The mean daily fluid volume decreased from 2500 mL to 1932 mL, aligning with the recommended target of 1800-2160 mL (Figure 2). 

## Discussion

Electrolyte analysis in the initial audit demonstrated inappropriate administration, with excessive sodium and chloride and insufficient potassium and glucose compared to the recommended daily requirement. This pattern reflects the overuse of isotonic fluids such as 0.9% sodium chloride (NaCl), which, while appropriate for resuscitation, are suboptimal for maintenance therapy due to their high sodium and chloride content and lack of potassium or glucose [9].

The re-audit showed improvement in compliance with standard recommendations. There was a marked reduction in sodium and chloride administration, along with an increase in potassium and glucose delivery. This improvement suggests that our multipronged intervention comprising default maintenance fluid changes, targeted teaching sessions, and visual awareness materials was effective in influencing prescribing practices. This outcome reflects improved prescribing behaviour, underpinned by a clearer understanding of the clinical rationale for maintenance fluid therapy.

Implementation challenges

Despite these encouraging results, the implementation phase presented several challenges that limited the full realisation and sustainability of the intervention.

Logistical Constraints

High-impact quality improvement projects (QIPs) are often resource-intensive, requiring both logistical and institutional support to ensure sustainability. One of the primary challenges we encountered was limited stock availability of the chosen hypotonic maintenance fluid, 0.45% NaCl with 0.15% KCl and 5% dextrose. Although clinically appropriate, this fluid was inconsistently available across surgical wards due to existing stock being prioritised for Variable Rate Intravenous Insulin Infusion [10]. Therefore, it is typically stocked in limited quantities reserved specifically for diabetic management. Our increased use of this solution for maintenance purposes inadvertently depleted central reserves, leading to supply shortfalls in both maintenance and diabetic management settings. This highlighted the unintended consequences of implementing change without parallel logistical planning.

Organisational Rigidity

In addition to logistical challenges, aspects of organisational rigidity also hindered the sustained implementation of the intervention. Organisational rigidity refers to resistance to change, often driven by limited access to relevant information or the absence of a culture that adapts effectively to external developments [11]. A notable example was prescriber inertia, where clinicians, particularly senior staff, reverted to familiar prescribing habits such as routinely selecting isotonic fluids. This reluctance to adopt new practices is well documented in the literature, especially in high-pressure clinical environments where time constraints tend to reinforce established routines over change [12]. Additionally, this inertia may be partly attributed to a desire to preserve professional autonomy and scepticism towards changes perceived as externally imposed [13].

Workforce Turnover and Team Instability

Frequent resident doctor rotations, variable shift patterns, and reliance on temporary or agency staff undermined the continuity of educational efforts [14]. Each new cohort of clinicians arrived with differing levels of awareness and training, necessitating repeated cycles of training and reinforcement. This fragmentation weakened the durability of the intervention and required ongoing engagement to mitigate regression to previous habits.

Interprofessional Dynamics

The response to the intervention was also shaped by interprofessional relationships. It was observed that staff tended to respond more positively when changes were introduced or endorsed by members of their own professional group. For instance, nursing staff were more receptive to guidance delivered by senior nurses, while doctors engaged more readily with consultant-led education [15]. Leveraging peer influence and leadership within each professional group is therefore critical to driving lasting behavioural change across multidisciplinary teams. 

Recommendations for sustainability

To overcome these barriers, several recommendations are proposed. Formal approval is required through clinical governance channels to designate this fluid as a standard maintenance option, alongside its existing role in VRIII. This should be supported by the development and ratification of a trust-wide IV fluid prescribing guideline that ensures consistent stocking, clear usage indications, and departmental accountability.

The project should be scaled across other departments and replicated in additional sites within the NHS Trust to ensure widespread impact. Finally, collaboration with the Postgraduate Medical Education team could enable integration of IV fluid prescribing education into the Foundation Year 1 induction programme, ensuring consistent knowledge among incoming doctors.

Empowering professional group leaders (e.g., consultants and senior nurses) to deliver peer-led training may also enhance engagement and reinforce a culture of evidence-based prescribing.

These structural changes are essential to embedding the improvement into routine practice. Only with clear policy, reliable availability of appropriate fluids, and sustained education can improvements in IV fluid prescribing be maintained across the healthcare system.

Limitations

This quality improvement project has several limitations that may affect the generalisability and interpretation of the findings. Firstly, the study was conducted at a single district general hospital, which may limit the applicability of the results to other institutions with differing patient populations, resource availability, and clinical practices. Secondly, the sample size was relatively small, which may not fully capture the variability of prescribing behaviours across other surgical cohorts or specialties. Data collection was limited to 24-hour periods, potentially underestimating cumulative fluid burden over the course of a patient’s hospital stay. While the intervention included educational components, the long-term retention of prescribing behaviours and the sustainability of practice change were not assessed beyond the immediate post-intervention audit. Future studies with larger, multicentre cohorts, longitudinal data collection, and longer follow-up periods are required to confirm the effectiveness and durability of these interventions across a broader context.

## Conclusions

Optimising IV maintenance fluid prescribing is critical to reducing fluid-related complications and improving surgical patient outcomes. This project demonstrated that targeted interventions can enhance adherence to national guidelines, promoting safer and more effective IV fluid management. Continued efforts, including guideline implementation, wider-scale audits, and improved education, are needed to sustain and further improve prescribing practices within the hospital and to promote safer IV fluid management on a national scale. In addition, embedding fluid stewardship into routine clinical practice and ensuring regular feedback to prescribers will be essential in driving long-term improvements. Future work could also focus on integrating decision-support tools into electronic prescribing systems to minimise variation and error. By combining education, system-level changes, and ongoing monitoring, hospitals can create a sustainable culture of safe and evidence-based IV fluid prescribing.
